# FAIM Is a Non-redundant Defender of Cellular Viability in the Face of Heat and Oxidative Stress and Interferes With Accumulation of Stress-Induced Protein Aggregates

**DOI:** 10.3389/fmolb.2020.00032

**Published:** 2020-02-27

**Authors:** Hiroaki Kaku, Thomas L. Rothstein

**Affiliations:** ^1^Center for Immunobiology, Western Michigan University Homer Stryker M.D. School of Medicine, Kalamazoo, MI, United States; ^2^Department of Biomedical Sciences, Western Michigan University Homer Stryker M.D. School of Medicine, Kalamazoo, MI, United States

**Keywords:** FAIM, cellular stress, protein aggregation, ubiquitin, cell death

## Abstract

A key element of cellular homeostasis lies in the way in which misfolded and dysfunctional proteins are handled. Cellular pathways that include proteasomal destruction and autophagic disposal are components of normal proteostasis. Here we report a novel molecule that plays a non-redundant role in maintaining homeostasis, Fas Apoptosis Inhibitory Molecule (FAIM). FAIM is highly conserved throughout evolution and bears no homology to any other protein. We found that FAIM counteracts heat and oxidative stress-induced loss of cell viability. FAIM is recruited to ubiquitinated proteins induced by cellular stress and the levels of stress-induced protein aggregates are much greater in FAIM-deficient cell lines. Primary fibroblasts from FAIM-deficient mice showed the same proteostasis deficits as cell lines. Administration of a mediator of oxidative stress to FAIM-deficient animals induced more ubiquitinated protein aggregates and more organ damage as compared to wild type mice. These results identify a completely new actor that protects cells against stress-induced loss of viability by preventing protein aggregation.

## Introduction

A key element of cellular homeostasis lies in maintaining proteostasis to avoid accumulation of dysfunctional proteins due to stress-related insults. Protein homeostasis is particularly critical for cells because cells are continuously exposed to cellular stress which causes protein misfolding (Chen et al., 2011; Diaz-Villanueva et al., 2015; Gandhi et al., 2019). Here we report a new mammalian protein, Fas Apoptosis Inhibitory Molecule (FAIM, also termed FAIM1), that protects cells from cellular stress. FAIM was originally cloned as a FAS antagonist in mouse primary B lymphocytes (Schneider et al., 1999). A subsequent study identified the alternatively spliced form, termed FAIM-Long (L) (Zhong et al., 2001), which has 22 additional amino acids at the N-terminus. Thus, the originally identified FAIM was renamed FAIM-Short (S) (Zhong et al., 2001). FAIM-L is expressed almost exclusively in the brain and in the testis whereas FAIM-S is ubiquitously expressed (Zhong et al., 2001). Recently, the *faim-Gm6432* gene, thought to be duplicated from the original *faim* gene, was identified in Muroidea rodents and its expression is limited to the testis (Qiu et al., 2013).

Intriguingly, *in silico* analysis indicates the existence of *faim* genes in the premetazoan genomes of single-celled choanoflagellates like *M. brevicollis* and *S. rosetta*, which is one of the closest living relatives of animals and a progenitor of metazoan life that first evolved over 600 million years ago (King et al., 2008; Fairclough et al., 2013). *S. rosetta* contains only 9411 genes, out of which two *faim* genes were found (Fairclough et al., 2013). This evidence suggests that the *faim* gene evolved much earlier than many other genes and domains found in multicellular organisms, including the death domain involved in animal cell apoptosis (Arvanitis et al., 2013; Zmasek and Godzik, 2013; Quistad and Traylor-Knowles, 2016), and implies that this gene may have another major function beyond apoptosis regulation. However, a lack of known consensus effector/binding motifs and even partial sequence homology of FAIM with any other protein has to date rendered it difficult to predict such functions (Schneider et al., 1999).

A series of overexpression studies demonstrated that FAIM produces resistance to FAS (CD95)-mediated apoptosis in B lymphocytes (Schneider et al., 1999), HEK293T cells (Li et al., 2014) and PC12 cells (Segura et al., 2007), enhances CD40-mediated NF-κB activation in B lymphocytes (Kaku and Rothstein, 2009), and induces neurite outgrowth in the PC12 cell line (Sole et al., 2004). Thus, FAIM expresses multiple activities related to cell death, signaling, and neural cell function. Nonetheless, the overarching physiological role of FAIM has remained unclear due to a lack of obvious phenotypic abnormalities of FAIM-deficient mice and cells.

The expression and evolution patterns of the *faim* and *faim-Gm6432* genes suggested that FAIM may be important for testicular functions (Qiu et al., 2013). Testicular cells are highly susceptible to heat shock (Durairajanayagam et al., 2015) and oxidative stress (Turner and Lysiak, 2008), which in turn suggested that FAIM might be involved in the cellular stress response. We therefore hypothesized that FAIM might regulate cellular stress response pathways in testicular cells or even in other cell types.

This led to the present study, reported here, indicating that FAIM fulfills the previously unknown role of protection against stress in various kinds of cell types. We found FAIM counteracts stress-induced loss of cellular viability. In this process, FAIM is recruited to ubiquitinated proteins. Importantly, more ubiquitinated, detergent-insoluble protein aggregates are accumulated in FAIM-deficient cells and tissues after cellular stress induction. These findings strongly suggest a novel, FAIM-specific role in holozoan protein homeostasis.

## Materials and Methods

### Ethics Statement

All mouse studies were performed at the Western Michigan University Homer Stryker M.D. School of Medicine campus in accordance with guidelines and protocols approved by the Institutional Animal Care and Use Committee (IACUC Protocols; Nos. 2016-006, 2016-009, and 2017-007).

### Antibodies

Rabbit anti-GFP, rabbit anti-ubiquitin, mouse anti-α-tubulin, goat anti-rabbit IgG-HRP-linked and horse anti-mouse IgG-HRP-linked antibodies were obtained from Cell Signaling Technology. Mouse anti-FLAG (M2) antibody and mouse anti-β-actin antibody were obtained from MilliporeSigma. Mouse anti-ubiquitin (UB-1) was obtained from Abcam. Affinity purified anti-FAIM antibody was obtained from rabbits immunized with CYIKAVSSRKRKEGIIHTLI peptide (located near the C-terminal region of FAIM) as previously described (Kaku and Rothstein, 2009).

### Plasmids

FLAG-tag-hFAIM-S expression vector was constructed using pCMV-(DYKDDDDK)-C (Takara) (cloned into EcoR- and Kpn- sites). Primers used for the cloning are shown in Supplementary Table S1. The insert was verified by sequencing (Genewiz). pSpCas9(BB)-2A-GFP (PX458) (Ran et al., 2013), plasmid #48138, was obtained from Addgene.

### Generation of FAIM-Deficient Mice

FAIM-deficient (KO) mice were generated in conjunction with the inGenious Targeting Laboratory. The target region, including the *faim* coding regions of exons 3–6 (9.58 kb), was replaced by sequences encoding eGFP and neomycin-resistance genes (Supplementary Figure S2A). The targeting construct was electroporated into ES cells derived from C57BL/6 mice. Positive clones were selected by neomycin and screened by PCR and then microinjected into foster C57BL/6 mice. Subsequent breeding with wild-type C57BL/6 mice produced F1 heterozygous pups. Offspring from heterozygous mice were selected using PCR. Mice were maintained on a C57BL/6 background. We performed genotyping PCR using genomic DNA from ear punches with a mixture of four primers to identify the wild-type allele and the mutant alleles, generating 514 and 389 bp DNA amplicons, respectively (Supplementary Figure S2B). Primers are shown in Supplementary Table S1. Mice were cared for and handled in accordance with National Institutes of Health and institutional guidelines. FAIM-KO mice were viable, developed normally and did not show any obvious phenotypic changes in steady state conditions (data not shown). The heterozygous intercrosses produced a normal Mendelian ratio of FAIM^+/+^, FAIM^+/–^, and FAIM^–/–^ mice. Mice at 7–12 weeks of age were used for all experiments.

### Cell Culture and Transfection

HeLa and GC-2spd(ts) cell lines were obtained from the American Type Culture Collection (ATCC). HeLa cells were cultured in DMEM medium (Corning) whereas GC-2spd(ts) cells were cultured in EMEM (Corning). Both DMEM and EMEM contained 10% FCS, 10 mM HEPES, pH 7.2, 2 mM L-glutamine and 0.1 mg/ml penicillin and streptomycin. Transfection was performed using Lipofectamine 2000 for GC2spd(ts) cells or Lipofectamine 3000 for HeLa cells, according to the manufacturer’s instructions (Themo Fisher Scientific). Primary fibroblasts were purified and cultured as previously described (Seluanov et al., 2010). Briefly, skin in the underarm area (1 cm × 1 cm) was harvested in PBS. The tissue was cut into 1 mm pieces. To extract cells, tissues were incubated at 37°C with shaking in 0.14 Wunsch units/ml Liberase Blendzyme 3 (MilliporeSigma) and 1 × antibiotic/antimycotic (Thermo Fisher Scientific) in DMEM/F12 medium (Corning) for 30–90 min until the medium became “fuzzy.” Tissues were washed with medium three times and then cultured at 37°C. After 7 days, cells were cultured in EMEM containing 15% FBS plus penicillin/streptomycin for another 7 days. Cells were used at this point for experiments.

### Generation of FAIM Knockout Cell Lines With CRISPR/Cas9

Guide RNA (gRNA) sequences for both human and mouse FAIM gene were designed using a CRISPR target design tool^1^ in order to target the exon after the start codon (Ran et al., 2013). Designed DNA oligo nucleotides are shown in Supplementary Table S1. Annealed double strand DNAs were ligated into pSpCas9(BB)-2A-GFP (PX458) vector (Addgene) at the Bpi1 (Bbs1) restriction enzyme sites using the “Golden Gate” cloning strategy. The presence of insert was verified by sequencing. Empty vector was used as a negative control. Transfection was performed using lipofection and a week after the transfection, eGFP^+^ cells were sorted with an Influx instrument (Becton Dickinson), and seeded into 96 well plates. FAIM knockout clones were screened by limiting dilution and western blotting.

### *In vitro* Cellular Stress Induction

To induce mild heat shock, cells in culture dishes were incubated in a water bath at 43°C for the indicated period (Wang et al., 2013). In some experiments, cells were recovered at 37°C after heat stress induction at 43°C for 2 h or more as previously described (Wang et al., 2013). To induce oxidative stress, menadione (MN) (MilliporeSigma), dissolved in DMSO at 100 mM, was added to medium at the indicated final concentration for 1 h. In oxidative stress experiments where cells were harvested at time points beyond 1 h with menadione, cells were washed once with medium and fresh medium (without menadione) was added to the cell culture as previously described (Koczor et al., 2009). Fibroblasts were treated with sodium arsenite (MilliporeSigma) at the indicated final concentration to induce oxidative stress. To induce FAS-mediated apoptosis in GC-2spd(ts), cells were cultured with 5 μg/ml anti-FAS antibody (clone; Jo2, BD Pharmingen) as previously described (Chandrasekaran et al., 2006).

### *In vivo* Mouse Stress Induction

Acute oxidative stress was induced by a single intraperitoneal injection of menadione (200 mg/kg in PBS) into mice (Hong et al., 2009; Liu et al., 2014), and then mice were euthanized 18 h after the injection. Spleens and livers were removed and protein was immediately extracted for western blotting analysis.

### Cell Viability Analysis With Flow Cytometry

Adherent cells were detached by Trypsin-EDTA. Adherent and floating cells were harvested and pooled, after which cells were resuspended in 2 μg/ml 7-aminoactinomycin D (7-AAD) (Anaspec). Cell viability was assessed using Gallios (Beckman Coulter) or Attune (Thermo Fisher Scientific) flow cytometers. Data were analyzed using FlowJo v9 or v10 software (FlowJo, LLC).

### Viability Analysis by Released LDH Detection

Following stress induction *in vitro* or *in vivo*, LDH released into the supernatant, or into the serum, from damaged cells was quantified using the Cytotox 96 Non-radioactive Cytotoxicity Assay (Promega). Serum samples were diluted in PBS (1:20).

### ALT Activity Assay

Following stress induction *in vivo*, serum was harvested and ALT levels were monitored using the ALT Activity Assay Kit (BioVision). OD at 570 nm (colometric) was detected with a Synergy Neo2 instrument. Serum samples were diluted in ALT assay buffer (1:5).

### Western Blotting

Cells were washed twice with PBS and lysed in RIPA lysis buffer (1% Nonidet P-40 (NP-40), 0.5% sodium deoxycholate, 0.1% SDS, 150 mM NaCl, 50 mM Tris–HCl (pH 8.0), 2 mM EDTA) containing supplements of 2 mM Na_3_VO_4,_ 20 mM NaF, and a protease inhibitor cocktail (Calbiochem) for 30 min on ice. In addition to the above supplements, 10 mM N-ethylmaleimide (NEM) (MilliporeSigma), 50 μM PR-619 (LifeSensors) and 5 μM 1,10-phenanthroline (LifeSensors) were added in the lysis buffer for ubiquitin detection by western blotting. Lysates were clarified by centrifugation at 21,100 × *g* for 10 min. Supernatants were used as RIPA-soluble fractions. The insoluble-pellets (the RIPA-insoluble fractions) were washed twice with RIPA buffer and proteins were extracted in 8M urea in PBS (pH 8.0). Protein concentrations were determined using the 660nm Protein Assay Reagent (Pierce). Protein samples in 1× Laemmli buffer with 2-ME were boiled for 5 min. Equal amounts of protein for each condition were subjected to SDS-PAGE followed by immunoblotting. Signals were visualized using a ChemiDoc Touch Imaging System (Bio-Rad) and Image Lab software (Bio-Rad).

### Immunoprecipitation

Cells expressing FLAG-tag proteins were lysed in 0.4% NP-40, 150 mM NaCl, 50 mM Tris–HCl (pH 8.0), 2 mM EDTA, 2 mM Na_3_VO_4,_ 50 mM NaF, and protease inhibitor cocktail for 30 min on ice. Lysates were clarified by centrifugation at 21,100 × *g* for 10 min. Equal amounts of protein for each supernatant were mixed with anti-FLAG M2 Magnetic Beads (MilliporeSigma) and incubated at 4°C under gentle rotation for 2 h. Beads were washed with lysis buffer four times and FLAG-tag proteins were eluted with 100 μg/ml 3× FLAG peptide (MilliporeSigma) two times. Eluates were pooled and western blotting was performed to detect FLAG-FAIM binding proteins.

### Filter Trap Assay (FTA)

WT and FAIM KO cells were incubated with or without menadione then harvested after the indicated period to detect ubiquitinated protein aggregates. Cells were washed with PBS and then lysed in PBS containing 2% SDS, 1 mM MgCl_2,_ protease inhibitor cocktail and 25 unit/ml Benzonase (MilliporeSigma). Protein concentrations were quantified using 660 nm Protein Assay Reagent with Ionic Detergent Compatibility Reagent (IDCR) (Thermo Fisher Scientific). Equal amounts of protein extracts underwent vacuum filtration through a pre-wet 0.2 μm pore size nitrocellulose membrane (GE Healthcare) for the detection of ubiquitinated protein aggregates using a 96 well format Dot-Blot apparatus (Bio-Rad). The membrane was washed twice with 0.1% SDS in PBS and western blotting using anti-ubiquitin antibody was carried out to detect aggregated proteins.

### *In situ* Proximity Ligation Assay (PLA)

Cells were cultured for 24 h on poly-L-lysine coated coverslips (Corning) in 24-well plates. Cells with or without cellular stress induction were fixed and permeabilized with ice-cold 100% methanol. PLA reaction was carried out according to the manufacturer’s instructions using Duolink *In Situ* Detection Reagents Orange (MilliporeSigma). ProLong Gold Antifade Reagent with DAPI (Cell Signaling Technology) was used to stain nuclei and to prevent fading of fluorescence. Fluorescence signals were visualized with a Nikon A1R^+^ confocal microscope (Nikon).

### Gene Expression Analysis by qPCR

Gene expression was assayed by real-time PCR. RNA was prepared from cells using the RNeasy mini kit (Qiagen), according to the manufacturer’s instructions. cDNA was prepared using iScript reverse transcription supermix (Bio-Rad). Gene expression was then measured by real-time PCR using iTaq SYBR Green (Bio-Rad) and normalized with GAPDH. Primer sequences are shown in Supplementary Table S1.

### Statistical Analysis

All quantitative data are expressed as mean ± SEM. ANOVA or, when appropriate, unpaired *t*-test was used for statistical determinations with GraphPad Prism 7 software. Values of *p* < 0.05 are considered statistically significant (^∗^*p* < 0.05, ^∗∗^*p* < 0.01 or ^∗∗∗^*p* < 0.001).

## Results

### FAIM KO Cells Are Susceptible to Heat/Oxidative Stress-Induced Cell Death

To test the activity of FAIM with respect to stress in testicular cells, we established a FAIM-deficient GC-2spd(ts) germ cell line by CRISPR-Cas9 excision and confirmed FAIM-deficiency by western blotting (Supplementary Figure S1A). FAIM KO and WT GC-2spd(ts) cells were cultured under stress conditions and cell viability was assayed by 7-AAD staining. After heat shock and after oxidative stress, cell viability in GC-2spd(ts) cells was markedly diminished in the absence of FAIM as compared to its presence (Supplementary Figures S1B,C). We did not observe a significant difference in FAS-induced cell death between FAIM-sufficient and FAIM-deficient cells (Supplementary Figures S1B,C).

**FIGURE 1:**
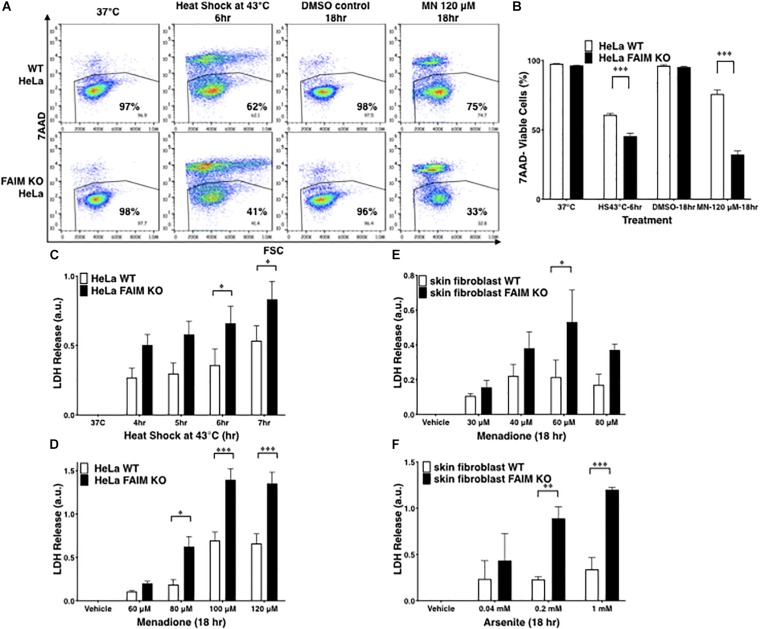
FAIM KO cells are susceptible to heat/oxidative stress-induced cell death. **(A)** WT and FAIM KO HeLa cells were incubated under stress conditions as noted for the indicated periods of time, and then stained with 7-AAD and cell viability was analyzed by flow cytometry. Representative flow data are shown. **(B)** A summary of pooled data from three independent experiments **(A)** is shown. **(C,D)** Cell viability was determined by supernatant LDH leaked from WT and FAIM KO HeLa cells upon heat shock **(C)** or upon menadione-induced oxidative stress **(D)**, as indicated. Pooled data from three independent experiments are shown. **(E,F)** Primary fibroblasts from WT and FAIM KO mice were subjected to menadione-induced **(E)** and arsenite-induced **(F)** oxidative stress, and cell viability was determined by supernatant LDH. Pooled data from three independent experiments are shown. All quantitative data are expressed as mean ± SEM. Two-way ANOVA was used to calculate *p*-values.

To exclude the possibility that FAIM protection is limited to germ cells, FAIM-deficient HeLa cells were generated with CRISPR-Cas9 (Supplementary Figure S1D). Similar to GC-2spd(ts) cells, FAIM-deficient HeLa cells were highly susceptible to stress-induced cell death (Figures 1A,B), to a greater extent than WT HeLa cells. To confirm these results with high-throughput methodology, we measured supernatant levels of lactate dehydrogenase (LDH) released from dead cells upon stress induction and again we found increased cellular disruption in the face of FAIM deficiency (Figures 1C,D).

To validate the role of FAIM in primary cells, we developed FAIM KO mice, in which the mouse *FAIM* gene (Supplementary Figures S2A,B) was disrupted and we confirmed by WB that FAIM protein was deficient in various tissues from FAIM^–/–^ mice (Supplementary Figure S2C). These mice were normal with respect to development, survival, and phenotype. They were not obese and thus differed from the FAIM KO mice constructed by Huo et al. (2009, 2016). We examined skin-derived fibroblasts which have been shown to be susceptible to menadione- (Shalini et al., 2012) and arsenite- (Harada et al., 2008; Kim et al., 2015) induced oxidative stress. Consistent with the cell line results, we found vulnerability to oxidative stress induced by menadione (Figure 1E) and by arsenite (Figure 1F) to be much greater in FAIM-deficient primary fibroblasts as compared to WT fibroblasts. In both cases LDH release increased with increasing dose except for menadione at 80 μM; this may have resulted from early rapid cell death at this high dose with loss of assayable LDH. Thus, data from three different cell types indicate that FAIM plays an essential role in protecting cells from heat and oxidative insults.

### Caspase-Dependent Apoptosis and ROS Production Are Normal in FAIM KO Cells Under Stress Conditions

Oxidative stress and heat shock induce caspase-dependent apoptosis via ROS generation (Slimen et al., 2014), which could play a role in stress-induced cell death that is affected by FAIM. To address this issue, we first evaluated ROS generation in FAIM-deficient and WT HeLa cells during oxidative stress, using the CellRox deep red staining reagent. We found no difference in stress-induced ROS, regardless of the presence or absence of FAIM (Supplementary Figure S3A). We then evaluated caspase activation under stress conditions, using the CellEvent caspase 3/7 detection reagent. We found that caspase 3/7 activity was not increased in FAIM-deficient HeLa cells (Supplementary Figure S3B).

To further evaluate stress-induced cell death, we separated cell death into caspase-dependent and caspase-independent forms. We pretreated cells with the pan-caspase inhibitor, Z-VAD-fmk peptide, before adding menadione, and then measured LDH release (Supplementary Figure S3C). Z-VAD-fmk has been reported to partially block menadione-induced cell death (Gerasimenko et al., 2002). We found menadione-induced LDH release was reduced to a small extent in both FAIM-deficient and FAIM-sufficient cells, resulting in similar levels of caspase-dependent apoptosis (Supplementary Figure S3D). Importantly, the increased LDH release induced by menadione in FAIM-deficient cells was for the most part resistant to caspase inhibition (Supplementary Figure S3E). Thus, menadione-induced cellular dysfunction, which is greatly magnified in the absence of FAIM, is largely caspase independent. In sum, there is no evidence that ROS/caspase-dependent apoptosis plays any role in the improved cellular viability produced by FAIM in the face of stress conditions.

### FAIM KO Cells Show a Normal Heat Shock Response

Heat shock proteins (HSPs) respond to stress conditions by upregulating expression (Richter et al., 2010). We examined HSP expression including HSPB1, HSPB5, and HSP70A1A in FAIM KO HeLa cells to determine if expression is upregulated by heat shock. We found that upregulation of these HSPs in FAIM KO HeLa cells were comparable with that in WT HeLa cells (Supplementary Figure S4), suggesting that the susceptibility of FAIM KO cells to cellular stress is not due to an impaired heat shock response.

### FAIM Is Recruited to the Complex Containing Ubiquitinated Proteins After Cellular Stress Induction

**FIGURE 2:**
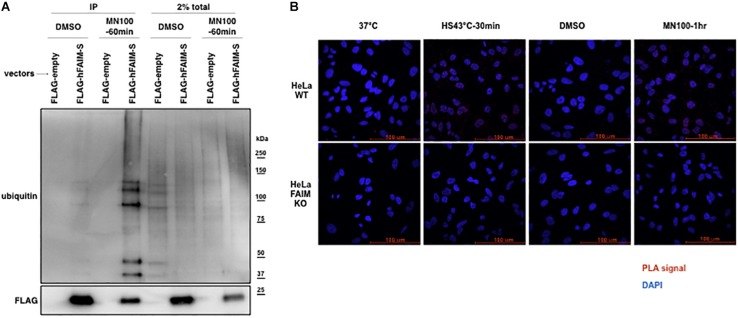
FAIM is recruited to ubiquitinated proteins after cellular stress induction. The interaction of FAIM and ubiquitinated proteins after cellular stress induction was assessed by co-immunoprecipitation **(A)** and *in situ* PLA **(B)**. (A) FAIM KO HeLa cells were transiently transfected with FLAG-tagged FAIM protein. Transfected FAIM KO HeLa cells were subjected to oxidative stress by incubation with menadione (MN, 100 μM) for 1 h, or were incubated with DMSO (the diluent for menadione), after which cells were harvested. Lysates were immunoprecipitated with anti-FLAG and subjected to SDS-PAGE and western blotted for ubiquitin. **(B)** FAIM KO HeLa cells and WT HeLa cells were subjected to heat shock (HS) at 43°C or oxidative stress (MN, 100 μM), as indicated, and then fixed and permeabilized, after which PLA reaction was carried out to detect proximity of FAIM and ubiquitin. Red dots indicate PLA positive signals and nuclei are stained blue with DAPI. Similar results were obtained from at least two independent experiments.

Stress-induced cellular dysfunction is often associated with the appearance of disordered and dysfunctional proteins that must be disposed of to maintain cellular viability. Stress-induced disordered proteins are tagged with ubiquitin for intracellular degradation via the proteasome system and the autophagic pathway (Lilienbaum, 2013; Ji and Kwon, 2017). To determine whether stress-induced loss of viability is associated with FAIM recruiting to ubiquitinated proteins, we again examined FAIM KO HeLa cells. FAIM KO HeLa cells were transfected with FLAG-tagged FAIM proteins and subjected to oxidative stress followed by anti-FLAG IP and western blotting for ubiquitin (Figure 2A). Separately, HeLa cells were subjected to heat shock and oxidative stress followed by PLA to detect close proximity of FAIM and ubiquitin (Figure 2B). Both Co-IP and PLA approaches demonstrated stress-induced interaction between FAIM and ubiquitinated protein. These data indicate that FAIM is recruited to the complex containing ubiquitinated proteins in response to cellular stress induction that, as noted above, is less deleterious to FAIM-sufficient HeLa cells as opposed to FAIM-deficient HeLa cells. Of note, we cannot rule out the possibility that FAIM binds aggregated proteins that are subsequently ubiquitinated.

### FAIM-Deficient Cells Accumulate Ubiquitinated, Aggregated Proteins in the Detergent-Insoluble Fraction After Stress Induction

**FIGURE 3:**
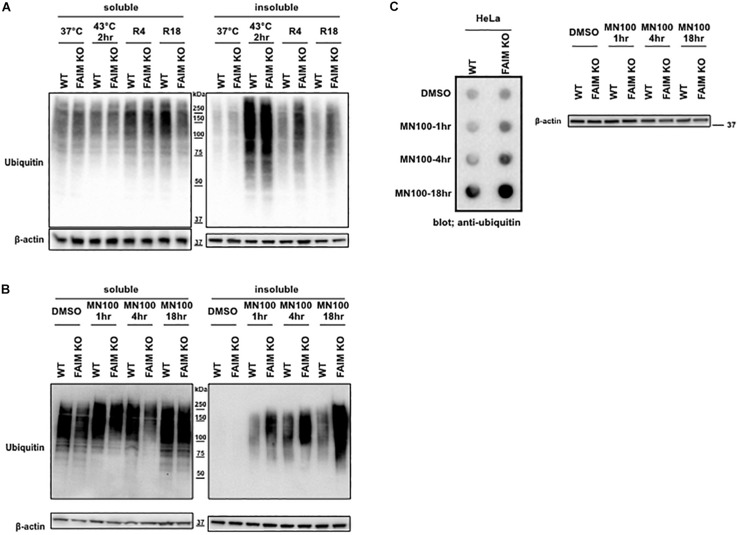
FAIM-deficient cells accumulate ubiquitinated, aggregated proteins in the detergent-insoluble fraction after stress induction. **(A)** WT HeLa cells and FAIM KO HeLa cells were incubated at 37°C or subjected to heat shock at 43°C for 2 h followed by recovery at 37°C for 4 h (R4) and for 18 h (R18). Cells were lysed and detergent soluble and detergent insoluble fractions were isolated. Equal amounts of protein for each fraction were analyzed by western blotting for ubiquitin and actin as a loading control. **(B)** WT HeLa cells and FAIM KO HeLa cells were incubated with menadione (MN) at 100 μM for the times indicated, or were incubated with DMSO vehicle. Cells were then handled as in panel **(A)**. **(C)** WT HeLa cells and FAIM KO HeLa cells were incubated with menadione (MN) at 100 μM for the indicated times, after which aggregated proteins were filter trapped and blotted with anti-ubiquitin. The same samples used in panel **(C)** were analyzed by western blotting for actin as a loading control.

The interaction between FAIM and ubiquitinated proteins induced by stress (Figure 2) suggests that impaired viability in stressed FAIM-deficient cells may be due to accumulation of cytotoxic, ubiquitinated protein aggregates that have overwhelmed disposal mechanisms. To determine if ubiquitinated protein aggregates increase after stress and do so disproportionately in the absence of FAIM, we assessed stress-induced accumulation of ubiquitinated proteins in FAIM KO HeLa cells vs. WT HeLa cells by western blotting. We found ubiquitinated proteins rapidly accumulated in detergent-insoluble fractions after heat shock for 2 h in both FAIM-sufficient and FAIM-deficient HeLa cells, however, the clearance of ubiquitinated proteins during the recovery period was slower in FAIM-deficient HeLa cells than FAIM-sufficient HeLa cells (Figure 3A). Furthermore, we also induced oxidative stress, which elicited milder, slower accumulation of protein aggregates in detergent-insoluble fractions than heat shock, and found that more ubiquitinated proteins in detergent-insoluble fractions were accumulated in FAIM-deficient HeLa cells compared to FAIM-sufficient HeLa cells (Figure 3B).

Next, to confirm that ubiquitinated proteins detected by western blotting in the detergent-insoluble fractions represent aggregated proteins, we performed filter trap assay (FTA) using total cell lysates from cells after oxidative stress. In this assay, large aggregated proteins are not able to pass though the 0.2 μm pore-sized filter and remain on the filter (Myeku et al., 2011). We observed that more aggregated proteins from FAIM-deficient HeLa cell lysates (Figure 3C) were trapped on the membrane during oxidative stress as compared to WT HeLa lysates. The same was true for primary mouse skin-derived fibroblasts from FAIM KO mice as compared to fibroblasts from WT mice (Supplementary Figures S5A,B). Thus, following stress, FAIM is recruited to the complex containing ubiquitinated proteins that, when excessive, accumulate in detergent-insoluble material, and accumulation of insoluble ubiquitinated proteins is much greater in the absence of FAIM. These results strongly suggest that FAIM is involved in the disposition of stress-induced aggregated proteins, and operates to divert such proteins from a pathway leading to insoluble protein aggregates.

### Ubiquitinated Protein Aggregates Accumulate in FAIM-Deficient Tissues Following Oxidative Stress *in vivo*

**FIGURE 4:**
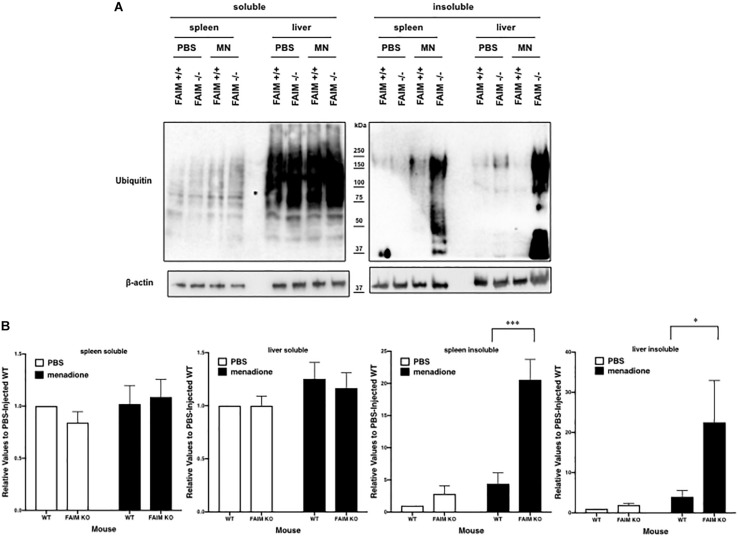
FAIM-deficient tissues accumulate ubiquitinated, aggregated proteins in the detergent-insoluble fraction after stress induction *in vitro*. **(A)** Spleen and liver tissues from FAIM KO mice and their littermate controls were collected 18 h after intraperitoneal administration of PBS or menadione (MN, 200 mg/kg). Tissue lysates were immediately extracted and protein samples were subjected to SDS-PAGE and western blotted for ubiquitin and actin as a loading control. Results are representative of at least six independent experiments. **(B)** Densitometry analysis is shown as fold change from control (PBS-injected WT). A summary of pooled data is shown. Data represent mean ± SEM. Two-way ANOVA was used to calculate *p*-values.

**FIGURE 5:**
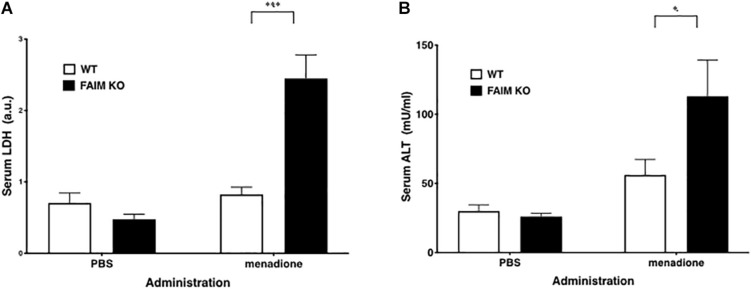
Serum LDH and ALT levels after stress induction are elevated in FAIM KO mice. **(A,B)** Serum samples obtained from mice treated as in Figure 4 were analyzed for content of LDH **(A)** and ALT **(B)**. Data represent mean ± SEM (*n* = 6). Two-way ANOVA was used to calculate *p*-value.

To demonstrate that FAIM-deficiency correlates with more insoluble, ubiquitinated protein upon cellular stress *in vivo*, we injected mice with menadione intraperitoneally, and assessed resultant tissue injury (Hong et al., 2009; Liu et al., 2014). Liver and spleen samples were collected 18 h after menadione administration into FAIM-deficient and littermate control FAIM-sufficient mice, and detergent-soluble and detergent-insoluble proteins were extracted. Similar to our *in vitro* experiments using HeLa cells and primary mouse fibroblasts, *in vivo* oxidative stress induced dramatically more ubiquitinated proteins in detergent-insoluble fractions from FAIM-deficient liver and spleen cells, as compared to liver and spleen cells from menadione-treated WT mice (Figures 4A,B). In accordance with these results, we found much higher levels of menadione-induced serum LDH (Figure 5A) and ALT (Figure 5B), which are signs of cell injury and death, in FAIM KO as compared to WT mice. These data indicate that FAIM plays a non-redundant role in preventing accumulation of insoluble, ubiquitinated, aggregated protein in stress-induced animals, and in protecting against cell death.

## Discussion

Although the FAIM gene arose in the genomes of the last common holozoan ancestor with a high level of homology among holozoan species, similar to house-keeping genes, its physiological function has been a long-standing enigma. Here, we have demonstrated that FAIM, originally thought of as a FAS-apoptosis inhibitor, plays an unexpected, non-redundant role in protection from cellular stress and tissue damage, leading to improved cellular viability (Figure 1 and Supplementary Figure S1). We have elucidated FAIM’s molecular mechanism by demonstrating that FAIM is recruited to, and binds, the complex containing ubiquitinated protein aggregates (Figure 2), rather than opposing caspase activity or dampening ROS generation (Supplementary Figure S3).

Cells and tissues are continuously subjected to environmental insults such as heat shock and oxidative stress, which cause accumulation of cytotoxic, aggregated proteins, leading to cell death. Organisms have evolved protective cellular mechanisms such as HSPs in order to prevent and counteract tissue and organ damage. FAIM has two characteristics that may contribute to its function. Firstly, the N-terminal region is relatively unstructured, according to NMR. It has been suggested that such disordered regions are important in binding aggregation prone targets (Hemond et al., 2009; Li et al., 2014). Further, FAIM protein sequences from choanoflagellate to human show significant underrepresentation of cysteine residues (only 1.4%) (Kriehuber et al., 2010). A reduced number of cysteines may be important to prevent unwanted crosslinking under oxidative conditions, enabling FAIM to resist denaturation and prevent protein aggregation induced by cellular oxidative stress (Kriehuber et al., 2010). Thus, in addition to proteasome degradation and autophagic disposal, our data support the role of FAIM as a new player that antagonizes cytotoxic protein aggregate formation and is not complemented by HSPs although we cannot exclude the possibility that FAIM plays a role in promoting the degradation of ubiquitinated protein aggregates by enhancing autophagic activity.

The aggregation of proteins into fibrillar high molecular-weight species is a hallmark of numerous human neurodegenerative disorders (Knowles et al., 2014). In the situation where overwhelming generation of misfolded or aggregated proteins due to cellular or aging stress occurs, these cytotoxic species must be degraded. However, in normal aged neuronal cells, autophagy-related genes are downregulated, leading to dysfunction of autophagy-mediated aggregate clearance (Lipinski et al., 2010). Further, proteasomal function has been reported to decline with age (Chondrogianni et al., 2014). Thus, in a situation of low autophagic and proteasomal activity, it might be speculated that the role of FAIM in preventing aggregation becomes more critical to maintaining proteostasis.

## Data Availability Statement

All datasets generated for this study are included in the article/Supplementary Material.

## Ethics Statement

The animal work was reviewed and approved by the Institutional Animal Care and Use Committee at Western Michigan University Homer Stryker MD School of Medicine (IACUC Protocols; Nos. 2016-006, 2016-009, and 2017-007).

## Author Contributions

HK designed and performed research, analyzed and interpreted data, and wrote the manuscript. TR analyzed and interpreted data, and wrote the manuscript.

## Conflict of Interest

The authors declare that the research was conducted in the absence of any commercial or financial relationships that could be construed as a potential conflict of interest.

## Footnotes

1 http://crispr.mit.edu
