# Supplementary material for: FAIM Is a Non-redundant Defender of Cellular Viability in the Face of Heat and Oxidative Stress and Interferes With Accumulation of Stress-Induced Protein Aggregates
Source: Front Mol Biosci. 2020 Feb 27;7:32. doi: 10.3389/fmolb.2020.00032 (PMC7056718; doi:10.3389/fmolb.2020.00032)
Supplement: Supplementary file 1 [file Table_1.DOCX]

**Title:** FAIM is a non-redundant defender of cellular viability in the face of heat and oxidative stress and interferes with accumulation of stress-induced protein aggregates

**Authors**: Hiroaki Kaku*, Thomas L. Rothstein*

**Affiliations**: Center for Immunobiology, Western Michigan University Homer Stryker MD School of Medicine, 1000 Oakland Drive, Kalamazoo, MI 49008. Tel: (269) 337-4380; e-mail: [hiroaki.kaku@med.wmich.edu](mailto:hiroaki.kaku@med.wmich.edu); [tom.rothstein@med.wmich.edu](mailto:tom.rothstein@med.wmich.edu)

Supplemental Materials

**Figure S1. FAIM KO cells are susceptible to heat/oxidative stress-induced cell death.**

**(A and D)** Western blot analyses of FAIM protein expression levels using cell lysates are shown for GC-2spd(ts) (A) and HeLa cells (D). **(B and C)** GC-2spd(ts) cells, were incubated under stress conditions as noted, for the indicated periods of time. Cell were also exposed to anti-FAS antibody for 24 hours. Cells were stained with 7-AAD and cell viability was analyzed by flow cytometry Representative flow data are shown in (B). A summary of pooled data from 3 independent experiments is shown in **(C)**. Data represent mean ± SEM. HS, heat shock; MN, menadione. Two-way ANOVA was used to calculate p-value.

**Figure S2. *faim KO m*ice lack exons 3-5**.

**(A)** Schematic representation of the targeting vector and the targeted allele of the mouse *faim* gene. **(B)** Genotype determination of *faim* mice by PCR. Multiplex PCR genotyping analyses for KO (389 bp) and WT (514 bp) *faim* genes were performed to confirm the genotypes of wild-type (^+/+^), heterozygous (^+/-^) and homozygous (^-/-^) mice. Representative genotyping results are shown. **(C)** FAIM protein expression was analyzed by western blotting using the indicated tissues from FAIM^+/+^ or FAIM^-/-^mice.

**Figure S3. Caspase-dependent Apoptosis and ROS production are normal in FAIM KO cells under stress conditions.**

**(A)** ROS production was measured by CellRox deep red staining reagent after oxidative stress induction. **(B)** Apoptosis induction was assessed by monitoring Caspase3/7 activation with CellEvent caspase3/7 detection reagent after oxidative stress induction as indicated. **(C, D and E)** Cell disruption was determined by LDH release with or without the pan-caspase inhibitor, Z-VAD-fmk, under oxidative stress conditions as indicated. Caspase-dependent cell death (D) and caspase-independent cell death (E) were calculated based on (C). A summary of pooled data from 3 independent experiments is shown. Data represent mean ± SEM. MN, menadione. Unpaired t-test was used to calculate p-value.

**Figure S4. FAIM KO cells show normal heat shock response.**

Human HSPB1 (HSP27), HSPB5 (αB-crystallin) and HSP70 A1A mRNA expression levels in HeLa cells and FAIM KO HeLa cells during heat shock conditions were analyzed by qPCR. Data are shown as fold change from no heat shock control. A summary of pooled data from 3 independent experiments is shown. Data represent mean ± SEM. R2; recovery at 37°C for 2 hours after heat stress at 43°C for 2 hours. R8; recovery at 37°C for 8 hours after heat stress at 43°C for 2 hours.

**Figure S5. FAIM-deficient primary fibroblasts accumulate ubiquitinated, aggregated proteins in the detergent-insoluble fraction after stress induction.**

**(A)** Primary mouse skin fibroblasts from WT and FAIM KO mice were incubated with menadione (MN) at 40 μM for the times indicated, or were incubated with DMSO vehicle. Cells were lysed and detergent soluble and detergent insoluble fractions were isolated. Equal amounts of protein for each fraction were analysed by western blotting for Ubiquitin, and actin as a loading control. **(B)** Primary mouse skin fibroblasts from WT and FAIM KO mice were incubated with menadione at 40 μM for the times indicated, or were incubated with DMSO vehicle. Aggregated proteins were filter trapped and blotted with anti-ubiquitin. Similar results were obtained from 2 independent experiments and representative data are shown in (A and B).

**Table S1: Primers/Oligonucleotides Used**

| Name | Sequence |
| --- | --- |
| mouse genotyping for WT allele fwd | ACGGATCTCGTAGCTGTTTGGGACG |
| mouse genotyping for WT allele rev | CCAGCGTGTACTCGTATGCGAAGCC |
| mouse genotyping for FAIM knockout allele fwd | CAGAAGAACTCGTCAAGAAGGC |
| mouse genotyping for FAIM knockout allele rev | CAAGCGAAACATCGCATCGAGCG |
| CRISPR-Cas9 oligo nucleotides for mouse FAIM fwd | CACCGTGACGGATCTCGTAGCTGTTTGG |
| CRISPR-Cas9 oligo nucleotides for mouse FAIM fwd | AAACAACAGCTACGAGATCCGTCAC |
| CRISPR-Cas9 oligo nucleotides for human FAIM fwd | CACCGACAGATCTCGTAGCTGTTTGGG |
| CRISPR-Cas9 oligo nucleotides for human FAIM fwd | AAACAAACAGCTACGAGATCTGTC |
| FAIM-S cloning into pCMV-(DYKDDDDK)-C fwd | ATATAGAATTCTAATGACAGATCTCGTAGCTGTTTGG |
| FAIM-S cloning into pCMV-(DYKDDDDK)-C rev | ATGGTACCACTTGCAATCTCTGGGATTTCT |
| sequence primer for pX-458 | TGGACTATCATATGCTTACCGTAACTTGAAAG |
| mouse HSP25 RT-qPCR fwd | ACTGGCAAGCACGAAGAAAG |
| mouse HSP25 RT-qPCR rev | AGGGAAGAGGACACTAGGGT |
| mouse HSP70 A1A RT-qPCR fwd | CAGTCCGACATGAAGCACTG |
| mouse HSP70 A1A RT-qPCR rev | CATCTTCGTCAGCACCATGG |
| mouse GAPDH RT-qPCR fwd | GTGCAGTGCCAGCCTCGTCC |
| mouse GAPDH RT-qPCR rev | CAGGCGCCCAATACGGCCAA |
| human HSP27 RT-qPCR fwd | ACATGAAGCACTGGCCTTTC |
| human HSP27 RT-qPCR rev | TCTCCTTCATCTTGGTCAGCAC |
| human HSP70 A1A RT-qPCR fwd | ACATGAAGCACTGGCCTTTC |
| human HSP70 A1A RT-qPCR rev | TCTCCTTCATCTTGGTCAGCAC |
| human GAPDH RT-qPCR fwd | CTGACTTCAACAGCGACACC |
| human GAPDH RT-qPCR rev | GTGGTCCAGGGGTCTTACTC |
